# Clinical Audit on Documentation of Anticipatory “Not for Resuscitation” Orders in a Tertiary Australian Teaching Hospital

**DOI:** 10.4103/0973-1075.78448

**Published:** 2011

**Authors:** Naveen Sulakshan Salins, Wendy Jansen

**Affiliations:** Consultant, Department of Integrative Oncology, HCG Enterprise Ltd-Bangalore Institute of Oncology, Bangalore, India; 1 Mary Potter Nursing Research Fellow, Discipline of Nursing. The University of Adelaide, Adelaide, Australia

**Keywords:** Audit, Documentation, Not for resuscitation

## Abstract

**Aim:**

The purpose of this clinical audit was to determine how accurately documentation of anticipatory Not for Resuscitation (NFR) orders takes place in a major metropolitan teaching hospital of Australia.

**Materials and Methods:**

Retrospective hospital-based study. Independent case reviewers using a questionnaire designed to study NFR documentation reviewed documentation of NFR in 88 case records.

**Results:**

Prognosis was documented in only 40% of cases and palliative care was offered to two-third of patients with documented NFR. There was no documentation of the cardiopulmonary resuscitation (CPR) process or outcomes of CPR in most of the cases. Only in less than 50% of cases studied there was documented evidence to suggest that the reason for NFR documentation was consistent with patient’s choices.

**Conclusion:**

Good discussion, unambiguous documentation and clinical supervision of NFR order ensure dignified and quality care to the dying.

## INTRODUCTION

Anticipatory Not for Resuscitation (NFR) order is a discussed, documented decision made in advance in patients with life-limiting illness where there is a plan to withhold active life-prolonging treatment in the event of an anticipated life-threatening situation. Discussion and documentation of NFR in any clinical setting should be guided by the following essential principles: a. Policy understanding b. Cardiopulmonary resuscitation (CPR) outcomes c. Person making the decision d. Person responsible for decisions made d. Family involvement e. Mandatory information that needs to be documented when documenting NFR f. Documentation of non-CPR measures that are appropriate for a given patient (IV antibiotics, fluids, feeding, pain relief, oxygen etc) g. Review of NFR decision.[1] The default position for patients in cardiac arrest is that CPR should always be commenced. However, in patients with life-limiting illness if there is a life-threatening situation during afterhours then the attending medical emergency team who may not be directly involved in the care need to have a clear directive about the patient’s resuscitation status and its accompaniments. This study helps to understand the contents, style and discussions during documentation of NFR orders in patients with advanced stages of life-limiting illness in an Australian teaching hospital.

## MATERIALS AND METHODS

### Design

This study is a retrospective evaluation of the accuracy of the documentation of NFR orders among patients who died at the Lyell McEwin Hospital, a university teaching hospital in South Australia, from 1 January 2007 to 31 December 2007. The palliative care Client Management Engine (CME) database was used to identify patients who died in the hospital during 2007. The CME system manages service provision, care planning, and measures data. All South Australian Palliative Care Services use the system by allowing comparison and assisting in smoother service provision. Once identified, all patients who died at home, nursing homes, other hospitals or other supported facilities were excluded from the study. One hundred and ninety-six patients who were referred to the palliative care service or were directly under palliative care died at the Lyell McEwin Hospital in the year 2007 and after randomization, 96 deceased files were eligible for review but only 88 files could be accessed from the archives at the point of study. This is most likely due to other audits taking place within the hospital, or the notes having been requested previously by other Medical Teams.

### Developing the questionnaire

The questionnaire was developed using the Australian Clinical Practice Guidelines for Communicating Prognosis and End-of-Life Issues with Adults in the Advanced Stages of a Life-Limiting Illness, and their Caregivers combined with Do Not Attempt Resuscitation Policy and Resuscitation Status and Medical Treatment Plan of LDS Hospital, School of Medicine, University of Utah.[2,3]

### Audit

Senior Palliative Care Registered Nurses from the Hospital’s Palliative Care Service completed all questionnaires. Their expertise allowed for the correct translation of data from patient case notes to audit criteria. All patient case notes were delivered from the Medical Records Department, auditors then completed the audit in one sitting, having access to a Palliative Medicine Specialist for complex entries. Following this, the Quality Improvement Manager for the Department of Medicine; Lyell McEwin Hospital processed the data using Microsoft Excel. Ethics approval was granted from The Central Northern Adelaide Health Service Ethics of Human Research Committee.

## RESULTS

Eighty-eight patient case notes were audited. The patients were between 27 and 101 years of age, the mean age being 70 years. Fifty-four patients (61.36%) were male and 34 (38.64%) were female.

Sixty-four patients (72.72%) had a major diagnosis of cancer and 24 patients (27.28%) had a non-malignant condition as their major diagnosis. Lung cancer was the most common cancer diagnosis (35.94% of cancers) and chronic lung disease was the commonest non-cancer diagnosis (33.33% of non-cancer).

Eighty-five patients (96.59%) had an anticipatory NFR order documented. The three patients who did not have an anticipatory NFR documented were not palliative care inpatients and two of them had a cancer diagnosis. Two patients who had anticipatory NFR orders documented underwent attempted resuscitation.

Among discussions regarding reason for documenting NFR are as follows. In 47.06% of cases the reason for documenting of NFR was that patients actively declined to have CPR in the event of a cardiopulmonary arrest. In 27.06% of patients, there was documented evidence to indicate that successful CPR is likely to be followed by a length and quality of life that would not be in the best interests of patients to sustain. Surrogate decision-making by the family member for those patients who were not mentally competent to make decisions were seen in 17.65% of patients [Table 1].

**Table 1:** Reasons for NFR discussion and documentation (*n* = 85)

Reasons for NFR discussion and documentation	Total (*n* = 85)
a. Patient’s existing condition indicates that effective CPR is unlikely to be successful	06 (7.06)
b. CPR is not in accord with the recorded, sustained wishes of the patient who is mentally competent	40 (47.06)
c. CPR is not in accord with a valid applicable Advance Directive	02 (2.35)
d. Successful CPR is likely to be followed by a length and quality of life that would not be in the patient’s best interests to sustain	23 (27.06)
f. Family member or proxy for patient with inadequate decision- making capacity aggress with NFR order	15 (17.65)

Figures in parenthesis are in percentage; NFR-not for resuscitation; CPR-Cardiopulmonary resuscitation

More than half (52.94%) had documented discussion about prognosis and documented evidence of discussion of diagnosis was noted only in 40.00% of cases. Two-thirds (69.41%) were offered palliative care or symptom control measures during the documentation of NFR. Documentation of CPR procedure or of the likelihood of CPR success was almost non-evident [Table 2].

**Table 2:** Content of NFR discussion and documentation (*n* = 85)

Details discussed and documented during the process of NFR documentation	Total (*n* = 85)
a. Diagnosis	34 (40.00)
b. Prognosis	45 (52.94)
c. CPR procedure	01 (01.17)
d. Likelihood of CPR success	02 (02.36)
e. Alternative to full CPR	03 (03.52)
f. Quality of life	06 (07.06)
g. Palliative care/symptom control measures	59 (69.41)

Figures in parenthesis are in percentage

Name of the doctor writing NFR order was documented only by 81.18% of cases and three doctors did not sign the NFR order. Date was evidenced in most of the documentation, however, time of entry was registered in only half of the documentation. Grade of the doctor writing NFR was documented in only 24.70% of cases. Only in a quarter there was translation of NFR documentation from medical notes to nursing notes. Clear usage of terminology during documentation was noted in most (88.23%) of the cases [Table 3].

**Table 3:** Style of NFR documentation (*n* = 85)

Essentials of NFR documentation	Total (*n* = 85)
a. Name of the doctor writing the entry	69(81.18)
b. Signature of the doctor	81(95.29)
c. Position of the doctor writing the entry	21(24.70)
d. Date of entry	83(97.64)
e. Time of entry	46(54.11)
f. Clear use of terminology	75(88.23)
g. Evidence of discussion with patients or relatives	74(87.06)
h. Identification stickers	80(94.12)
i. Nursing staff aware of NFR decision (as evidenced by the nursing notes)	23(27.06)

Figures in parenthesis are in percentage

Consultants were directly involved in NFR decision-making in 32.94% of cases and in 66.66% of cases there is documented evidence to suggest that consultants have reviewed the patient within 48 h of NFR documentation by the junior doctor. There is no documented evidence to suggest that consultants have changed the NFR order written by the junior doctors.

Among the patients documented as NFR, 44.70% of patients received IV fluids and 28.23% of patients received IV antibiotics. Oxygen use was seen in half of the cases studied and most of the patients received pain relief. Two patients needing non-invasive ventilation had motor neuron disease [Table 4].

**Table 4:** Specific treatments offered to patients documented as NFR

Treatment offered	Total (*n* = 85)
a. IV fluids	38 (44.70)
b. Parenteral nutrition	1 (1.18)
c. Blood transfusion	7 (8.23)
d. IV antibiotics	24 (28.23)
e. Oxygen	41 (48.23)
f. Pain relief	79 (92.94)
g. Treatment of specific biochemical abnormalities	4 (4.70)
h. Other active treatment	8 (9.41)
i. Non-Invasive Ventilation	1 (1.18)

Figures in parenthesis are in percentage

There was documented evidence to say that 32 (37.65%) patients did not have the ability to make decisions, however, only five (5.88%) had an advanced medical directive. There was evidence of family involvement in NFR decision-making in 87.05% of cases.

## DISCUSSION

Among the 88 patients studied, the majority had a documented NFR order and among the documented NFR orders, nearly 50% of patients made a voluntary decision to not undergo resuscitation and in one-third of cases it was not medically appropriate to initiate resuscitation. Surrogate decision-making by the family was seen in less than 20% of cases. The role of next of kin or family is to reflect the patient’s view about end of life. However, if the patient is unable to make a competent decision then they cannot demand resuscitation when effective CPR is unlikely to be successful or successful CPR is likely to be followed by a length and quality of life that would not be in the best interests of the patient to sustain.[4] Decisions to withhold life-sustaining treatment are made in two different situations. In the first, treatment is withheld from an actively dying person whose existing condition indicates that effective CPR is unlikely to be successful or successful CPR is likely to be followed by a length and quality of life that would not be in best interests of the patient to sustain. In the second, the decision is hypothetical, to withhold treatment if the patient should develop a life-threatening condition.[5]

CPR is a form of intensive and invasive treatment associated with high mortality. Compared to other treatments this intensive treatment is poorly discussed and documented.[6] A study reported that elderly patients who first chose to be resuscitated, almost half changed their opinion after they received more detailed information about the possibility of surviving.[7] Often patients and patients’ relatives participate in decisions about resuscitation and end of life at a time of great emotional distress. In a short period of time, they are expected to digest and evaluate complex medical information and make decisions about themselves or for their loved ones. Therefore, prior knowledge, information and media portrayal strongly influence the decision-making.[8] It is important for the physicians to explain the process, clinical accompaniments and aftermath including intubation, mechanical ventilation, artificial feeding, hydration, supplemental oxygen and pharmacological agents. Therefore, decision of instituting CPR is not a single ethical decision but a number of choices either bundled together or spread over a period of time.[9]

Patient autonomy should be the cornerstone in deciding about the patient’s resuscitation status. Accurate information about the condition, prognosis, and nature of the proposed intervention, alternatives, risks and benefits may enable the patients to make better decisions about resuscitation and end of life.[10] In 50% of cases there was documentation to suggest diagnosis and prognosis were discussed. CPR and its accompaniments were hardly discussed and palliative care was offered in two-thirds of cases.

The study has pointed out a few common errors usually made while documenting an “NFR” order. These errors could create ambiguous situations usually afterhours when the patient is attended by a different healthcare professional who is unaware of the patient management goals. The documentation of NFR should include date and time, name, grade, signature, reason for NFR decision, evidence of discussion with the family, awareness among nursing and allied health staff about the decision and a clear review time. The default position for patients in cardiac arrest is that CPR should always be commenced.

As far as possible the senior doctors in the team should undertake decisions of “NFR”. If undertaken by a junior staff member then this should be revisited by a senior staff member within a reasonable timeframe so that the ultimate responsibility of this onerous and distressing task is shared.[11] This study shows that even after patients were documented “NFR” they were offered all possible ward measures. In patients where CPR is inappropriate they should not fear that they are abandoned. They should be reassured that they will not be deprived of symptom control measures, comfort, pain relief and dignity at the end of life. In some circumstances giving IV antibiotics, IV fluids, oxygen, blood transfusion and nutrition is appropriate provided it adds to symptom control and quality of life.

Documentation of NFR should clearly reflect the circumstances around which the decisions to forgo life-sustaining treatments are made. A good structured documentation increases the likelihood of a good process and good outcome. Good documentation is probably the easiest and safest way of informing nursing, allied health staff, medical emergency team and doctors on call after hours about the NFR decision. Without such information appropriate care of the patient cannot be ensured.[12] It is impractical to conclude that junior doctors should not make NFR decisions. Appropriate CPR decisions should be made at the earliest to avoid unnecessary CPR attempts in patients where they are clearly not indicated. It is advisable to consult the senior doctors in the team to help them discuss NFR decision or confirm their decision. Consultants need to review the decisions made by the junior doctors and subsequently at regular intervals.[13]

Documenting the care provided to the patients and families during the process of NFR decision-making will help us to understand how the decisions were made, indicates the care provided and quality of care. A good discussion may have taken place and good care provided, but the audit can only conclude what is provided based on the accuracy of the documentation.[14] Once the decision of NFR is made it is imperative that we document the rationale for the decision. Hospital policy and preprinted NFR forms increases the likelihood of doctors documenting NFR orders.[15]

## CONCLUSION

The following were the conclusions of this study

- Junior doctors need guided education on structure and style of documentation of NFR
- Need for better documentation and uniform style of documentation
- Need for hospital NFR policy and staff awareness about hospital NFR policy
- Introduction of standardized NFR forms to overcome barriers in documentation
